# Viable but nonculturable bacteria and their resuscitation: implications for cultivating uncultured marine microorganisms

**DOI:** 10.1007/s42995-020-00041-3

**Published:** 2020-05-13

**Authors:** Xiao-Hua Zhang, Waqar Ahmad, Xiao-Yu Zhu, Jixiang Chen, Brian Austin

**Affiliations:** 1 College of Marine Life Sciences and Institute of Evolution & Marine Biodiversity, Ocean University of China, Qingdao, 266003 China; 2 Laboratory for Marine Ecology and Environmental Science, Qingdao National Laboratory for Marine Science and Technology, Qingdao, 266071 China; 3 School of Petrochemical Engineering, Lanzhou University of Technology, Lanzhou, 730050 China; 4 Frontiers Science Center for Deep Ocean Multispheres and Earth System, Ocean University of China, Qingdao, 266100 China; 5 Institute of Aquaculture, University of Stirling, Stirling, FK9 4LA Scotland, UK

**Keywords:** VBNC, Cultivating the uncultured, Resuscitation, Marine microorganisms

## Abstract

Culturing has been the cornerstone of microbiology since Robert Koch first successfully cultured bacteria in the late nineteenth century. However, even today, the majority of microorganisms in the marine environment remain uncultivated. There are various explanations for the inability to culture bacteria in the laboratory, including lack of essential nutrients, osmotic support or incubation conditions, low growth rate, development of micro-colonies, and the presence of senescent or viable but nonculturable (VBNC) cells. In the marine environment, many bacteria have been associated with dormancy, as typified by the VBNC state. VBNC refers to a state where bacteria are metabolically active, but are no longer culturable on routine growth media. It is apparently a unique survival strategy that has been adopted by many microorganisms in response to harsh environmental conditions and the bacterial cells in the VBNC state may regain culturability under favorable conditions. The resuscitation of VBNC cells may well be an important way to cultivate the otherwise uncultured microorganisms in marine environments. Many resuscitation stimuli that promote the restoration of culturability have so far been identified; these include sodium pyruvate, quorum sensing autoinducers, resuscitation-promoting factors Rpfs and YeaZ, and catalase. In this review, we focus on the issues associated with bacterial culturability, the diversity of bacteria entering the VBNC state, mechanisms of induction into the VBNC state, resuscitation factors of VBNC cells and implications of VBNC resuscitation stimuli for cultivating these otherwise uncultured microorganisms. Bringing important microorganisms into culture is still important in the era of high-throughput sequencing as their ecological functions in the marine environment can often only be known through isolation and cultivation.

## Introduction

Culturing has been an important feature of microbiology since the landmark work of Robert Koch in the nineteenth century. The dogma is that cultures are comprised of living bacterial cells and therefore, their presence on laboratory media is reflective of viability. However, the proviso is that these organisms need to be able to grow on the available laboratory media. Unfortunately, there is not a single medium that permits the growth of all microorganisms. Therefore, recovery of culturable bacteria reflects the availability of suitable nutrients and the adoption of appropriate incubation regimes. The inevitable outcome is that only a small proportion of marine bacteria are culturable in the laboratory. Certainly, novel techniques have been developed, such as dilution to extinction, involving the use of filtered autoclaved seawater. This approach permitted the recovery of bacteria that could grow only in oligotrophic (very low nutrient) media and did not initially produce visible colonies. These organisms were truly obligate oligotrophs (Schut et al. 1993). However, many taxa have never been grown on artificial media (e.g. Fehr et al. 2013), for example “*Candidatus*”, but they may still have great importance in ecology and as the cause of disease in aquatic organisms. Multiple species of “*Candidatus*” have been described on the basis of DNA sequences and a few characteristics without the availability of pure cultures. For example, “*Candidatus* Syngnamydia venezia” has been reported as the causal agent of epitheliocystis in broad nosed pipefish (*Syngnathus typhle*; Fehr et al. 2013). *“Candidatus* Halichondribacter symbioticus” was reported as a sponge symbiont of *Halichondria panicea* with unknown function (Knobloch et al. 2020). Other organisms appear to cease culturability and the viable but nonculturable (VBNC) state is a typical example.

**Fig. 1:**
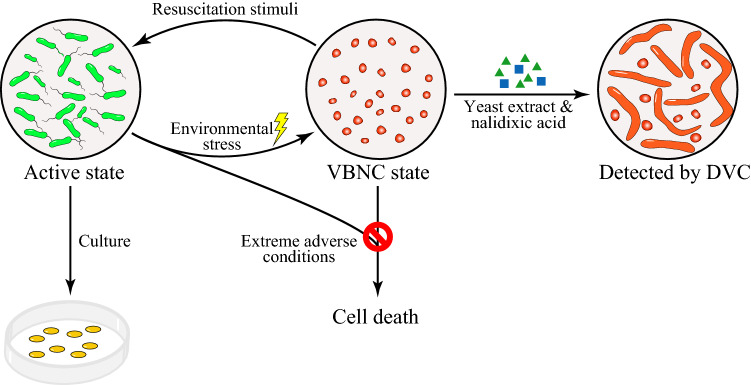
The life cycle of VBNC cells. VBNC refers to a physiological state where bacteria are metabolically active, but are no longer culturable on conventional growth media. It is a survival strategy adopted by many bacteria in response to harsh environmental conditions, and the VBNC cells may return to culturable state under favorable conditions

The VBNC state was first described by Xu et al. (1982), who found that an exponentially growing culture of *Vibrio cholerae* or *Escherichia coli*, subjected to incubation in a nutrient-free microcosm (e.g., sterile natural or artificial seawater free of nutrient) at low temperature (4 °C), exhibited a decline in culturability on conventional culture media under normal culture conditions. However, a portion of the non-culturable population remained viable when they were detected by the direct viable count (DVC) procedure developed by Kogure et al. (1979). Specifically, these VBNC cells were metabolically active and had the ability to elongate in the presence of nutrients, namely yeast extract and an inhibitor of cell division, i.e. nalidixic acid or cephalexin, but could not develop into visible colonies on conventional solid media (Fig. 1). This was the first attempt to distinguish viability from culturability, as cell viability was typically evaluated by the ability to produce visible colonies on solid media or turbidity in broth, respectively (Colwell and Grimes 2000; Pinto et al. 2015). Since then, the VBNC state has been studied extensively and demonstrated to be a unique survival strategy occurring over a wide range of Gram-negative bacteria, fewer Gram-positive bacteria, as well as some fungal species (Oliver 2010; Pinto et al. 2015). Cells enter the VBNC state in response to a variety of environmental stresses, which initiate a complex series or cascade of cellular events (Oliver 2016; Oruno et al. 2019). The bacterial cells in the VBNC state may return to a metabolically active and culturable state under appropriate circumstances (Fig. 1; Colwell and Grimes 2000; Dong et al. 2019; Pinto et al. 2015). However, care needs to be taken to ensure that apparent resuscitation does not reflect the growth of a low number of residual culturable cells that may have persisted in the environment or experimental system.

In the natural environment, microorganisms are threatened by a variety of stresses and therefore, certain strategies need to be employed that allow tolerance against conditions that are harmful to growth. The ability to enter the VBNC state is advantageous for the long-term survival of bacteria (Pinto et al. 2015), otherwise, these environmental stresses could potentially kill entire populations. These apparently dormant cells may later resuscitate when the stresses are relieved or when cells receive signals exhibiting favorable environmental conditions (Oliver 2016; Pinto et al. 2015). The survival of the fish pathogen *Streptococcus parauberis* was studied in seawater and sediments revealing culturability for ~ 1 month and ~ 6 months, respectively. Slightly higher survival occurred at 6 °C rather than 22 °C. During this period of culturability, metabolism declined. However, direct counts indicated that the total number of cells remained high even after culturability could not be detected. Afterwards, the addition of nutrients led to the return of culturable cells. Thus, it was reasoned that *Str. parauberis* could enter the VBNC state but this was reversible (Curras et al. 2002).

In this review, we focus on the issues correlated with bacterial culturability, the diversity of bacteria that enter into the VBNC state, environmental challenges that induce the VBNC state, conditions that help in resuscitation from the VBNC state, and the implications of resuscitation stimuli for cultivating the previously uncultured marine microorganisms.

## Issues associated with bacterial culturability

There are a number of explanations for the inability to culture bacteria in the laboratory, and they are listed below:

### Lack or excess of essential nutrients

For example, *Renibacterium salmoninarum*, which is the cause of bacterial kidney disease in salmonids, has an obligate requirement for cysteine as L-cysteine hydrochloride. Some essential nutrients may be provided by the growth of other organisms, i.e. satellitism (Austin and Austin 2016; Evelyn et al. 1989). Excessive quantities of nutrients inhibit oligotrophs, which thrive in low nutrient environments (Schut et al. 1993).

### Lack of appropriate osmotic support

Osmotically-fragile cells, i.e. spheroplasts (Takayanagi et al. 2016) and L-forms, need specialized media, incorporating sucrose and horse serum, to enable the development of very small colonies, which could be buried into the surface of the media. L-forms have been reported with *Aeromonas salmonicida*, which is the causal agent of furunculosis and ulcer disease in fish (McIntosh and Austin 1990) and possibly *Ren. salmoninarum* (Hirvela-Koski et al. 2006). Moreover, seawater systems, which were found to be devoid of culturable *Aer. salmonicida* using conventional plating methods*,* contained cells that passed through the pores of 0.22 µm filters, and produced colonies on a specialized L-form medium, i.e. L-F medium (Effendi and Austin 1991). It is possible that more wild strains in natural environments are sensitive to osmotic pressure.

### The lack of appropriate incubation conditions

Along with appropriate media, it is essential for incubation regimes to reflect the original ecological environment from which the organisms have been recovered. Attention needs to be given to the incubation temperature [psychrophilic (Showalter and Deming 2018), mesophilic or thermophilic], duration (many organisms are slow growing, and may need several weeks to develop visible growth), atmosphere (aerobic, micro-aerophilic or anaerobic) and pressure (deep-sea bacteria will inevitably require barophilic conditions if they are to grow at all). In addition, there may be a need for an appropriate surface on which the organisms can produce biofilms.

### Low growth rate

Slow-growing bacteria, such as *Ren. salmoninarum*, may be outcompeted on laboratory media by faster-growing aerobic heterotrophs unless active steps are taken to inhibit the unwanted organisms; this includes the use of selective isolation procedures involving disinfectants, including chlorhexidine gluconate (Nakashima et al. 2007) or antibiotics (Austin and Austin 2016). The ecological theory of “*K-*strategy” and “*r-*strategy” could also explain the competition between slow- and fast-growing bacteria; *K*-strategy slow growers have a stable existence in their habitat, *r-*strategy fast growers respond rapidly to nutrient flushing (Janssen 2009). Oligotrophic media could be used to culture the slow-growing bacteria.

### Development of micro-colonies

If culturability is akin to the development of visible colonies then limited growth leading to micro-colonies poses a dilemma for the study of VBNC. Such micro-colonies may not be seen by the naked eye (Torrella and Morita 1981) and the organism could be mistakenly thought to be uncultured. The search for micro-colonies would require the use of specialist sensitive methods, for example on-chip microscopy (Jung and Lee 2016). The reasons for the development of micro-colonies could include the exhaustion of key nutrients, the accumulation of potentially toxic metabolites or an issue with overcrowding.

### The existence of ultramicrobacteria

Many bacteria, which are often the dominant component of the microbiota in the marine environment, exist as or develop into extremely small cells, which are capable of passing through the pores of 0.22 µm pore size filters (Boenigk et al. 2004; Mukhanov et al. 2016; Obayashi and Suzuki 2019). These cells may be referred to as the ultramicrobacteria (Schut et al. 1993) for which culturing is not always possible. The cells may well be in a state of starvation (Fegatella and Cavicchioli 2000; Haller et al. 2000; Obayashi and Suzuki 2019) and being actively grazed by flagellates (Boenigk et al. 2004). Metabolic activity, including protease activity, has been described, indicating that the cells were viable (Obayashi and Suzuki 2019). These small cells have been linked with multiple taxa by 16S rRNA gene sequencing, and include *Pseudoalteromonas*, *Vibrionaceae* representatives and *Erythrobacter*/*Erythromicrobium/Sphingomonas* (Vybiral et al. 1999).

### The presence of senescent cells

Cells which are senescent and/or damaged, may require special techniques for their recovery. For example, pre-incubation in liquid media may lead to improved culturability rather than plating directly onto solid media (Olson 1978). It is possible that the liquid medium enables the recovery and/or repair of damaged cells, i.e. the cells need to adjust to the new environment (Rolfe et al. 2012).

### The presence of VBNC cells

**S**ome cells in the natural environment may be dormant and do not grow on laboratory media without a means of reactivation. The best studied examples of dormancy involve endospores, which are produced in some Gram-positive bacteria and are regarded as important survival structures particularly in marine sediments (Volpi et al. 2017; Wormer et al. 2019). However, in the marine environment, many Gram-negative cells have also been associated with dormancy, i.e., in the VBNC state (Kaprelyants et al. 1993; Xu et al. 1982). The VBNC state of bacteria is a classic example regarding the influence of bacterial physiological status on cultivation success (Roszak and Colwell 1987). Since most marine microorganisms live in oligotrophic and challenging natural environments surrounded by biological competitors, it is hypothesized that a considerable proportion of marine microbial communities may be in the VBNC state (Bodor et al. 2020). Thus, VBNC cells could constitute a huge reservoir of bacteria, which cannot be cultured easily with ordinary cultivation methods. Therefore, the resuscitation of VBNC microorganisms may act as an important means for cultivating previously uncultured (i.e. previously unsuccessfully cultivated) microorganisms.

## Diversity of bacteria entering the VBNC state

**Fig. 2:**
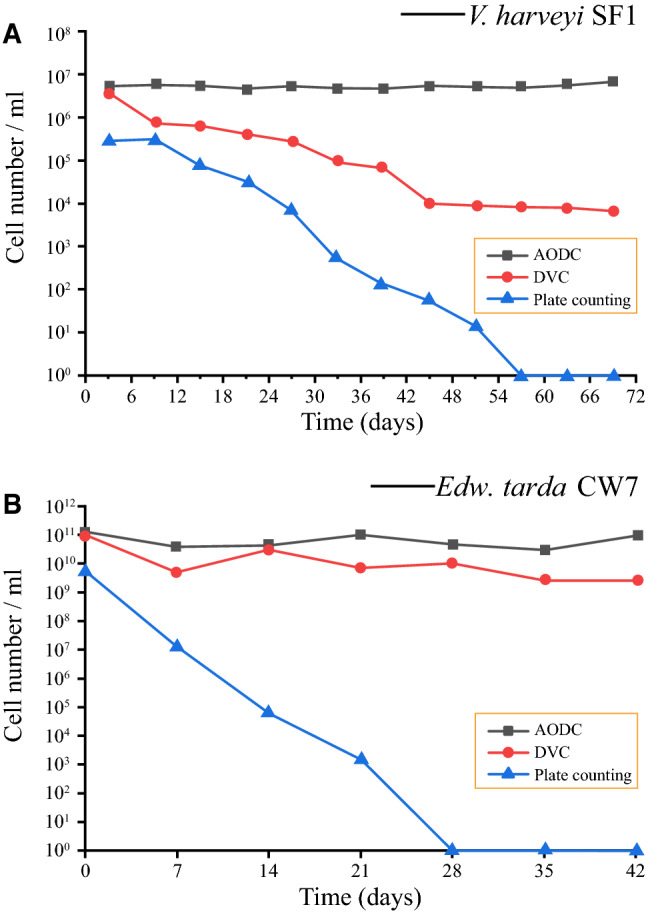
Entry of *Vibrio harveyi* and *Edwardsiella tarda* into the VBNC state at 4 °C as determined by AODC, DVC and plate counting methods. **a** *V. harveyi* SF1 in a seawater microcosm (redrawn from Sun et al. 2008); **b** *Edw. tarda* CW7 in an artificial seawater microcosm (redrawn from Du et al. 2007a)

After the initial description for *V. cholerae* and *Esc. coli* by Xu et al. (1982), VBNC cells were discovered among a wide range of bacteria (at least 50 genera and 101 species of bacteria; Table 1). The list includes a variety of important human pathogens, including *Burkholderia pseudomallei*, *Campylobacter jejuni*, pathogenic *Esc. coli*, *Helicobacter pylori*, *Klebsiella pneumoniae*, *Legionella pneumophila*, *Listeria monocytogenes*, *Mycobacterium tuberculosis*, *Pseudomonas aeruginosa*, *Salmonella enterica*, *V. cholerae*, and *Yersinia pestis* (Table 1). This list also includes many marine bacteria, including *Vibrio* species (i.e., *V. alginolyticus*, *V. anguillarum*, *V. cincinnatiensis*, *V. fischeri*, *V. harveyi*, *V. parahaemolyticus* and *V. vulnificus*) and *Edwardsiella tarda* (Table 1; Fig. 2). Subsequently, the VBNC state has also been found in a number of eukaryotes, most notably the yeasts *Saccharomyces cerevisiae*, *Brettanomyces bruxellensis* and *Cryptococcus neoformans* (Table 1).

**Table 1:** Bacterial and fungi species reported to enter into the VBNC state in different taxa (updated and modified from Oliver 2005, 2010 and Pinto et al. 2015)

**Bacteria** (50 genera, 101 species)	
***Proteobacteria*** (38 genera, 75 species) ***Alphaproteobacteria*** (6 genera, 8 species) *Acetobacter aceti* (Pinto et al. 2015) *Agrobacterium tumefaciens* (Oliver 2005) *Methylocella tundrae* (Misra et al. 2012) *Methylocystis hirsuta* (Misra et al. 2012) *Methylocystis parvus* (Misra et al. 2012) *Rhizobium leguminosarum* (Oliver 2005) *Rhizobium meliloti* (Oliver 2005) *Sinorhizobium meliloti* (Oliver 2005) ***Betaproteobacteria ***(5 genera, 6 species) *Acidovorax citrulli* (Kan et al. 2019) *Alcaligenes eutrophus* (Oliver 2005) *Burkholderia cepacia* (Oliver 2005) *Burkholderia pseudomallei* (Oliver 2005) *Cupriavidus metallidurans* (Giagnoni et al. 2018) *Ralstonia solanacearum* (Oliver 2005) ***Gammaproteobacteria*** (23 genera, 53 species) *Acinetobacter calcoaceticus* (Pinto et al. 2015) *Aeromonas hydrophila* (Oliver 2010) *Aeromonas salmonicida* (Oliver 2005) *Citrobacter freundii* (Pinto et al. 2015) *Edwardsiella tarda* (Du et al. 2007a, b) *Enterobacter aerogenes* (Oliver 2005) *Enterobacter agglomerans* (Pinto et al. 2015) *Enterobacter cloacae* (Oliver 2005) *Erwinia amylovora* (Oliver 2010) *Escherichia coli* (Oliver 2005) *Francisella tularensis* (Oliver 2005) *Legionella pneumophila* (Oliver 2005) *Methylocaldum gracile* (Misra et al. 2012) *Methylococcus capsulatus* (Misra et al. 2012) *Methylomicrobium alcaliphilum* (Misra et al. 2012) *Methylomonas methanica* (Misra et al. 2012) *Methylosarcina fibrata* (Misra et al. 2012) *Methylosinus sporium* (Misra et al. 2012) *Methylosinus trichosporium* (Misra et al. 2012) *Pasteurella piscicida* (Oliver 2005) *Pseudomonas aeruginosa* (Oliver 2005) *Pseudomonas fluorescens* (Oliver 2005) *Pseudomonas putida* (Oliver 2005) *Pseudomonas syringae* (Oliver 2005) *Salmonella bovismorbifican* (Abdallah et al. 2007) *Salmonella enterica* (Oliver 2005) *Salmonella enteritidis* (Oliver 2005) *Salmonella montevideo* (Davies and Evison 1991) *Salmonella oranienburg* (Davies and Evison 1991) *Salmonella typhi* (Cho and Kim 1999) *Salmonella typhimurium* (Davies and Evison 1991) *Serratia marcescens* (Oliver 2005) *Shigella dysenteriae* (Oliver 2005) *Shigella flexneri* (Oliver 2005) *Shigella sonneii* (Oliver 2005) *Vibrio alginolyticus* (Oliver 2010)	***Gammaproteobacteria*** (continued) *Vibrio anguillarum* (Oliver 2005) *Vibrio campbellii* (Oliver, 2005) *Vibrio cholerae* (Oliver 2005) *Vibrio cincinnatiensis* (Zhong et al. 2009) *Vibrio fischeri* (Oliver 2005) *Vibrio harveyi* (Oliver 2005) *Vibrio mimicus* (Oliver 2005) *Vibrio natriegens* (Oliver 2005) *Vibrio parahaemolyticus* (Oliver 2005) *Vibrio proteolytica* (Oliver 2005) *Vibrio salmonicida* (Hoff 1989) *Vibrio vulnificus* (Oliver 2005) *Xanthomonas axonopodis* (Oliver 2010) *Xanthomonas campestris* (Oliver 2005) *Yersinia enterocolitica* (Smith et al. 1994) *Yersinia pestis* (Pinto et al. 2015) ***Epsilonproteobacteria ***(4 genera, 8 species) *Arcobacter butzleri* (Pinto et al. 2015) *Campylobacter coli* (Oliver 2005) *Campylobacter jejuni* (Oliver 2005) *Campylobacter lari* (Oliver 2005) *Helicobacter pylori* (Oliver 2005) *Klebsiella aerogenes* (Oliver 2005) *Klebsiella planticola* (Oliver 2005) *Klebsiella pneumoniae* (Oliver 2005) ***Actinobacteria*** (5 genera, 12 species) *Arthrobacter albidus* (Su et al. 2011) *Arthrobacter crystallopoietes* (Ensign 1970) *Bifidobacterium animalis* (Pinto et al. 2015) *Bifidobacterium lactis* (Pinto et al. 2015) *Bifidobacterium longum* (Pinto et al. 2015) *Micrococcus flavus* (Byrd et al. 1991) *Micrococcus luteus* (Kaprelyants et al. 1994) *Mycobacterium bovis* (Lim et al. 1999) *Mycobacterium smegmatis* (Nikitushkin et al. 2012) *Mycobacterium tuberculosis* (Gample et al. 2019) *Rhodococcus biphenylivorans* (Su et al. 2015) *Rhodococcus rhodochrous* (Oliver 2005) ***Bacteroidetes (***1 genus, 1 species**)** *Cytophaga allerginae* (Oliver 2005) ***Firmicutes*** (6 genera, 13 species) *Enterococcus faecium* (Oliver 2005) *Enterococcus faecalis* (Oliver 2005) *Enterococcus hirae* (Oliver 2005) *Lactobacillus brevis* (Liu et al. 2018) *Lactobacillus lactis* (Oliver 2005) *Lactobacillus lindneri* (Pinto et al. 2015) *Lactobacillus paracollinoides* (Pinto et al. 2015) *Lactobacillus plantarum* (Oliver 2005) *Listeria monocytogenes* (Oliver 2005) *Oenococcus oeni* (Pinto et al. 2015) *Staphylococcus aureus* (Pasquaroli et al. 2013) *Streptococcus faecalis* (Byrd et al. 1991) *Streptococcus pyogenes* (Trainor et al. 1999)

**Fungi: Yeast** (7 genera, 7 species)	
*Brettanomyces bruxellensis* (Willenburg and Divol 2012; Capozzi et al. 2016)	*Rhodotorula mucilaginosa* (Divol and Lonvaud-Funel 2005)
*Candida stellata* (Divol and Lonvaud-Funel 2005)	*Saccharomyces cerevisiae* (Divol and Lonvaud-Funel 2005; Salma et al. 2013)
*Cryptococcus neoformans* (Hommel et al. 2019)	*Zygosaccharomyces bailii* (Divol and Lonvaud-Funel 2005)
*Dekkera bruxellensis* (Barata et al. 2008)

Bacterial species reported to enter the VBNC state are phylogenetically distributed: *Alphaproteobacteria* (6 genera, 8 species), *Betaproteobacteria* (5 genera, 6 species), *Gammaproteobacteria* (23 genera, 53 species), *Epsilonproteobacteria* (4 genera, 8 species), *Bacteroidetes* (1 genus, 1 species), *Actinobacteria* (5 genera, 12 species) and *Firmicutes* (6 genera, 13 species) (Table 1). Most of these (76 species) are Gram-negative bacteria (affiliated to the phyla *Proteobacteria* and *Bacteroidetes*), although 25 species comprised Gram-positive non-sporulating bacteria (affiliated to phyla *Actinobacteria* or *Firmicutes*). Some other Gram-positive bacteria, notably *Bacillus* and *Clostridium* species, could form endospores, which is the first reported bacterial survival state (Hutchison et al. 2014). In this connection, cysts comprise another survival state that may be observed in some Gram-negative bacteria (e.g. *Azotobacter* spp.).

The list of microbial species currently discovered to enter the VBNC state mainly reflects the research interest of scientists, which includes pathogen taxa (i.e., pathogens for human, plants and aquatic animals), those associated with food safety or environmental applications, rather than the actual existence of this survival process in natural microbial communities (Colwell and Grimes 2000). It may be assumed that the VBNC response is a universal process for microorganisms and may occur in a wide range of microbial taxa. Moreover, the initial concept of VBNC was narrow and based on already cultivatable microorganisms. In fact, VBNC may be a suitable term for defining the yet to be cultured microorganisms, which were viable in natural environments but non-culturable in routine growth media. Considering the vast quantity of microorganisms in the ocean, many of the uncultured microorganisms could be in the VBNC state.

## Physiological features and detection of the VBNC bacteria

**Fig. 3:**
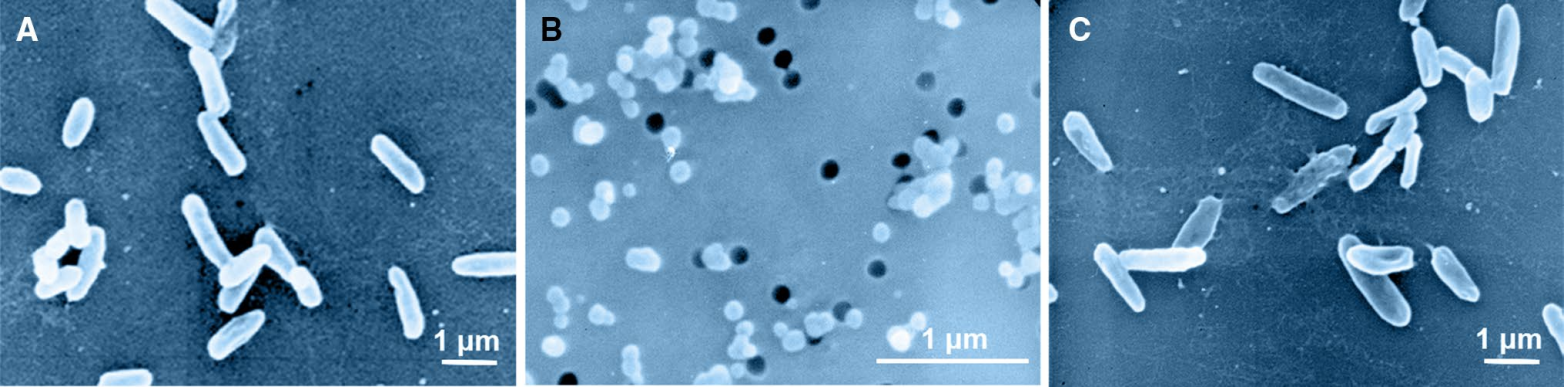
Morphological characteristics of *Vibrio harveyi* SF1 analyzed with a scanning electron microscope. **a** Normal cells; **b** VBNC cells; **c** Resuscitated cells

In the VBNC state, bacteria adopt lower growth rates and reduced levels of metabolism, e.g., there is a slowing down of the respiration rate, nutrient transport, and macromolecular synthesis. Many VBNC bacterial species decrease cell size, such as forming coccoid-shaped cells with enlarged periplasmic space (Fig. 3). The decreased surface/volume ratio may help bacteria to reduce their energy requirement (Bodor et al. 2020; Pinto et al. 2015). Often, VBNC bacteria retain their cell integrity and potential replication capabilities (Oliver 2016; Pinto et al. 2015). In addition, VBNC cells usually contain reduced concentrations of cytoplasm, total proteins and membrane fatty acids. However, VBNC cells contain relatively high ATP levels, and exhibit high membrane potential and increased O-acetylation and cross-linking in peptidoglycan cell walls (Oliver 2016; Pinto et al. 2015). Moreover, VBNC cells show decreased superoxide dismutase activity, and increased oxidative damage. Because of these changes, VBNC cells have enhanced resistance to antibiotics and physical and chemical stresses than do culturable cells. Furthermore, the potential for virulence among VBNC pathogens is unclear. Some VBNC pathogens are unable to cause diseases until they regain culturability. Conversely, others remain potentially pathogenic as they continue expressing toxins (Dong et al. 2019).

Detection in the absence of culturing initially focused on microscopy (e.g. the DVC procedure developed by Kogure et al. 1979), the LIVE/DEAD Baclight assay (evaluating cell viability based on cytoplasmic membrane integrity, with two fluorescent stains propidium iodide and SYTO 9; Boulos et al. 1999) but has since progressed to molecular methodologies, including loop-mediated isothermal amplification (LAMP) (Cao et al. 2019; Zhong et al. 2016). Both Cao et al. (2019) and Zhong et al. (2016) compared real time PCR (qPCR) and real time LAMP (qLAMP) in combination with propidium monoazide to detect VBNC cells of *V. parahaemolyticus* in seafood including shrimp, with the latter (in combination with propidium monoazide) being quicker and more sensitive.

## Mechanism of induction into the VBNC state

Since the first report of the VBNC state, a variety of factors (e.g., physical, chemical and biotic environmental parameters) that can initiate the cascade of cellular events leading to the VBNC state have been reported. The physical factors include high/low temperature, high/low salinity (osmotic stress), sub-optimal pH, sub-optimal redox conditions, sunlight, irradiation, drying, pulsed electric field and high-pressure stress. The chemical factors include nutrient starvation, antibiotic pressure, food preservatives, disinfectants (i.e., chlorination), nitrite, heavy metals, organic pollutants or exposure to other toxic compounds (Gample et al. 2019; Oliver 2005, 2010, 2016; Pinto et al. 2015). In addition, biotic factors may also be inducers of the VBNC state. For example, the protozoan *Acanthamoeba castellanii* was found to induce *Aer. hydrophila* into the VBNC state (Rahman et al. 2008), whereas the VBNC cells of the coral pathogen, *V. shiloi*, were reported to be associated with a marine fire worm (*Hermodice carunculata*; Sussman et al. 2003). In another case, the culture supernatant of the amoeba *Hartmannella vermiformis* induced the VBNC state of *Leg. pneumophila* (Buse et al. 2013). This was considered to be caused by nutrient depletion resulting from the animal growth as well as the presence of potentially harmful metabolic products in the medium. However, the environmental stresses inducing the VBNC state seem to vary between studies (Pinto et al. 2015). One possible explanation may be that the culture conditions investigated were always complicated, with several possible stresses interacting with each other. Many stresses, such as sub-optimal temperature, nutrient, salinity, pH, dissolved oxygen, and irradiation, may affect the viable process and lead to the VBNC state of the cells.

Since the first report of VBNC, many studies have focused on explaining the mechanism of VBNC formation (Oliver 2016; Pinto et al. 2015). However, there is still very little information on the genetic mechanisms behind the VBNC process (Trevors 2011). The environmental stresses that can induce the VBNC state may give clues to the underlying genetic regulation of VBNC cells.

One explanation of VBNC formation is that when actively growing microorganisms face a sudden shock, such as shortage of nutrients, change of pH, or the presence of harmful metabolites, it leads to the decoupling of growth from metabolism. Consequently, cells may suffer a burst of oxidative metabolism, which will accumulate peroxides and other free radicals within cells (Munn 2020). Microorganisms may avoid this occurrence if they are induced to make changes to protect the DNA, proteins, and other essential components in the cells. The shock of the sudden transfer of cells into a rich medium when they are still in the process of adaptation to life in the oligotrophic aquatic environment could otherwise result in sudden death (Munn 2020). The inability of VBNC cells to detoxify lethal free radicals either induced by the cells themselves or present in culture medium is one of the main reasons for the non-culturability. This process may be due to the repression of periplasmic catalase, which breaks down toxic peroxide (Munn 2020). As a result, several proteins have been shown to play a significant role in the formation of VBNC cells; these include superoxide dismutase (SodA), catalases KatA and KatG, RNA polymerase sigma S (RpoS), alkyl hydroperoxide reductase subunit C (AhpC), sensory histidine kinase (EnvZ), and a LysR-type transcriptional regulator (OxyR) (Dong et al. 2019).

## The resuscitation factors of VBNC cells

Despite VBNC cells typically having low levels of metabolic activity, through specific treatments, many cells are able to revert to a metabolically active and culturable state when cultured on conventional bacteriological media. The process of VBNC cells recovering to culturability is termed resuscitation (Colwell and Grimes 2000; Oliver 2010). Since the VBNC state is triggered by the environmental stresses mentioned above, eliminating these stresses may help reversion to culturability. Nevertheless, the resuscitation of some species through simply reversing the adverse stress is not always successful. It should be noted that the diversity of VBNC bacteria (50 genera, 101 species) is much higher than that of resuscitation (less than 20 species), mostly due to the lack of knowledge on the underlying mechanisms of this process. However, many conditions that promote the restoration of culturability have been identified, including physical stimuli (e.g., upshifting of temperature), chemical stimuli (e.g., pyruvate, glutamate, amino acids, Tween 20, vitamins, metal chelating agents or siderophore, and quorum sensing signal molecules), active proteins (e.g., Rpfs, YeaZ and catalase), or host associated stimuli (Table 2). The resuscitation process differs among different bacterial taxa, and may be initiated by several stimuli. The specific conditions that help in resuscitation from the VBNC state are described in more detail below.

**Table 2:** Resuscitation promoting factors for VBNC cells

Resuscitation promoting factors	Bacterial species tested (References)
**Physical stimuli**	
Temperature upshift	*Aeromonas hydrophila* (Maalej et al. 2004)
	*Escherichia coli* (Pinto et al. 2011)
	*Vibrio parahaemolyticus* (Wong et al. 2004)
	*V. vulnificus* (Oliver et al. 1995)
	*V. alginolyticus* (Du et al. 2007b)
Temperature upshift in the presence of yeast extract, Tween 20, vitamin B or catalase	*Edwardsiella tarda* (Du et al. 2007a)
	*V. alginolyticus* (Du et al. 2007b)
	*V. cincinnatiensis* (Zhong et al. 2009)
	*V. harveyi* (Sun et al. 2008)
Heat shock in rich culture media	*Salmonella enterica* (Gupte et al. 2003)
**Chemical stimuli**	
Sodium pyruvate	*Salmonella enteritidis* (Morishige et al. 2013)
	*Legionella pneumophila* (Ducret et al. 2014)
Glutamate	*Leg. pneumophila* (Ducret et al. 2014)
Gluconate	*Cupriavidus metallidurans* (Giagnoni et al. 2018)
Amino acids	*Esc. coli* (Pinto et al. 2011)
Rich culture media	*Arcobacter butzleri* (Fera et al. 2008)
	*Enterococcus faecalis* (Lleo et al. 1998, 2001)
	*Enterococcus hirae* (Lleo et al. 2001)
	*Esc. coli* (Ozkanca et al. 2009; Pinto et al. 2011)
Vitamins	*V. cincinnatiensis* (Zhong et al. 2009)
	*V. harveyi* (Sun et al. 2008)
Tween 20	*Sal. enterica* (Zeng et al. 2013)
Gas mixture	*Campylobacter jejuni* (Bovill and Mackey 1997)
Chelator	*Pseudomonas aeruginosa* (Dwidjosiswojo et al. 2011)
Siderophore	*Esc. coli* (Lewis et al. 2010)
	*Micrococcus luteus* (Lewis et al. 2010)
Quorum sensing molecules	*Esc. coli* (Liu et al. 2009)
	*V. vulnificus* (Ayrapetyan et al. 2014)
**Active proteins**	
Resuscitation promoting factor Rpf	*Mic. luteus* (Mukamolova et al. 2002)
	*Sal. enterica* (Panutdaporn et al. 2006)
Resuscitation promoting factor like protein YeaZ	*V. parahaemolyticus* (Aydin et al. 2011a, b)
	*V. harveyi* (Li et al. 2017)
Catalase	*Esc. coli* (Gourmelon et al. 1994)
	*Sal. enterica (*Zeng et al. 2013)
**Host associated stimuli**	
Protozoan	*Leg. pneumophila* (Garcia et al. 2007; Steinert et al. 1997)
Rabbit ileal loop	*V. cholerae* (Colwell et al. 1985)
Mouse model	*Vibrio vulnificus* (Oliver and Bockian 1995)
	*Cam. jejuni* (Cappelier et al. 1999)
Embryonated egg model	*Edw. tarda* (Du et al. 2007a)
	*Listeria monocytogenes* (Guillou et al. 2008)
	*Cam. jejuni* (Cappelier et al. 2007)

### Physical stimuli

The most common factor inducing the VBNC state for bacteria (e.g., *Vibrio* species and many other genera) is low temperature (Colwell and Grimes 2000; Oliver 2010). Many studies have shown that a temperature upshift is sufficient to allow resuscitation from the VBNC state induced by the low temperature (e.g., Du et al. 2007a, b; Gupte et al. 2003; Pinto et al. 2015; Wong et al. 2004). In this regard, climate change may well be accelerating *Vibrio* resuscitation rates (Oliver 2016). In addition, temperature upshift in the presence of chemical stimuli, such as yeast extract, Tween 20, vitamin B or catalase, were also useful in resuscitating VBNC cells (e.g., Du et al. 2007a, b; Sun et al. 2008; Zhong et al. 2009).

### Chemical stimuli

Sodium pyruvate has been reported as one of the principal promoters of resuscitating VBNC cells, with its function as a reactive oxygen scavenger, or so-called antioxidant, as well as a carbon source (Ducret et al. 2014; Vilhena et al. 2019). Sodium pyruvate can restore the biosynthesis of DNA, proteins and other macromolecules, thus resuscitating VBNC cells to a culturable state (Morishige et al. 2013; Vilhena et al. 2019). The VBNC cells of the human pathogen *Leg. pneumophila* could be resuscitated in culture media with other reactive oxygen scavengers, such as glutamate (Ducret et al. 2014). Also, gluconate could induce the resuscitation of the soil-borne organism *Cupriavidus metallidurans* from the VBNC state to a cultural state (Giagnoni et al. 2018).

Combinations of a variety of amino acids, such as asparagine, glutamine, methionine, serine and threonine, in the basal minimal medium were shown to effectively support the transition of *Esc. coli* VBNC cells (Pinto et al. 2011). Rich culture media and vitamins may resuscitate cells of many bacterial species from the VBNC state (Fera et al. 2008; Lleo et al. 1998, 2001; Ozkanca et al. 2009; Pinto et al. 2011; Sun et al. 2008; Zhong et al. 2009). However, the exact substance in rich culture media that is vital to the resuscitation process remains unclear.

The addition of 3% (*v*/*v*) Tween 20 allowed the VBNC coccoid cells of *Sal. enterica* serovar Typhi to regain culturability, again within 24–48 h, and the resuscitated cells remained virulent as evidenced by animal infectivity experiments (Zeng et al. 2013). Moreover, the dormancy of the *Cam. jejuni* VBNC cells, caused by low oxygen availability, could be restored in the presence of a microaerobic gas mixture (Bovill and Mackey 1997). The dormancy of *Pse. aeruginosa* VBNC cells, caused by the presence of toxic concentrations of copper ions, could be resuscitated by the addition of the copper-ion chelator diethyldithiocarbamate. Then, the resuscitated cells showed cytotoxicity to the eukaryotic Chinese Hamster Ovary cell line (Dwidjosiswojo et al. 2011). Siderophores promote cell division. Furthermore, Lewis et al. (2010) showed that the siderophores from *Esc. coli* and *Micrococcus luteus* could be used as growth factors for uncultured bacterial strains.

Quorum sensing (QS) signal molecules have been reported to correlate with the resuscitation of cells from the VBNC state (Ayrapetyan et al. 2014; Liu et al. 2009), probably correlated with the ability to increase antioxidative capacity (Mesrop et al. 2014). QS is a cell-to-cell communication system in bacteria that works through the production, release, detection and group-level response to signaling molecules, called autoinducers (Papenfort and Bassler 2016). *Esc. coli* O157:H7 was reported to be resuscitated by the autoinducer 2 (A1-2) that was produced during biofilm formation process in a serum-based medium (Liu et al. 2009). In addition, A1-2 could reverse the VBNC state in *V. vulnificus* (Ayrapetyan et al. 2014). Furthermore, it has been reported that QS could trigger catalase expression leading to resuscitation of *Salmonella typhimurium* VBNC cells independent of the OxyR regulon (Liao et al. 2019). These phenomena suggest that QS exerts an important role in the resuscitation process.

### Active proteins

In Gram-positive bacteria, a group of extracellular bacterial proteins, known as resuscitation-promoting factors (Rpfs), were shown to have an important role in promoting resuscitation of VBNC cells (Mukamolova et al. 1998, 2002; Pinto et al. 2015). Rpfs have been reported in a variety of Gram-positive bacteria, including *Mic. luteus, Corynebacterium* spp., *Lis. monocytogenes*, *Mycobacterium* spp., *Streptomyces* spp., *Tomitella biformata* and *Sal. enterica* serovar Typhimurium (Pinto et al. 2015). The Rpfs from different bacterial species may have different structures and activities but all share a conserved domain of ~ 70 amino acids and possess a lysozyme-like activity (e.g., peptidoglycan lytic or muralytic activity). The mechanism of Rpfs on resuscitating VBNC cells probably centers on the ability to cleave cell wall compositions, thereby discharging the lysis products. These may function as signaling molecules for growth initiation or modifying the mechanical properties of the cell wall to enable cell division (Kana and Mizrahi 2010; Keep et al. 2006).

In Gram-negative bacteria, the resuscitation-promoting like factors belong to an obviously different protein class, named YeaZ, which allows cells to survive in and exit from the VBNC state. Panutdaporn et al. (2006) reported that YeaZ from *Sal. typhimurium* could promote resuscitation of VBNC cells of *Sal. enterica* serovar Oranienburg. In addition, the expression of a YeaZ homologue was essential for the survival of *Esc. coli* cells (Handford et al. 2009). Furthermore, YeaZ of *V. parahaemolyticus* acted as a classic actin-like nucleotide-binding protein, and exerted an important role in reverting the *V. parahaemolyticus* VBNC cells (Aydin et al. 2011a, b). However, the underlying molecular mechanism of YeaZ in the resuscitation of VBNC cells remains unknown.

Catalase is the hydrogen peroxide degradation protein that may promote resuscitation of the VBNC state of *Esc. coli* as induced by phototoxicity of visible light (Gourmelon et al. 1994). Catalase is effective in reducing phototoxicity by eliminating hydrogen peroxide, thiourea, a hydroxyl radical scavenger and desferrioxamine B. The addition of 1% (*v*/*v*) catalase allowed the VBNC cells of *Sal. enterica* to return to culturability (Zeng et al. 2013).

### Host associated stimuli

**Fig. 4:**
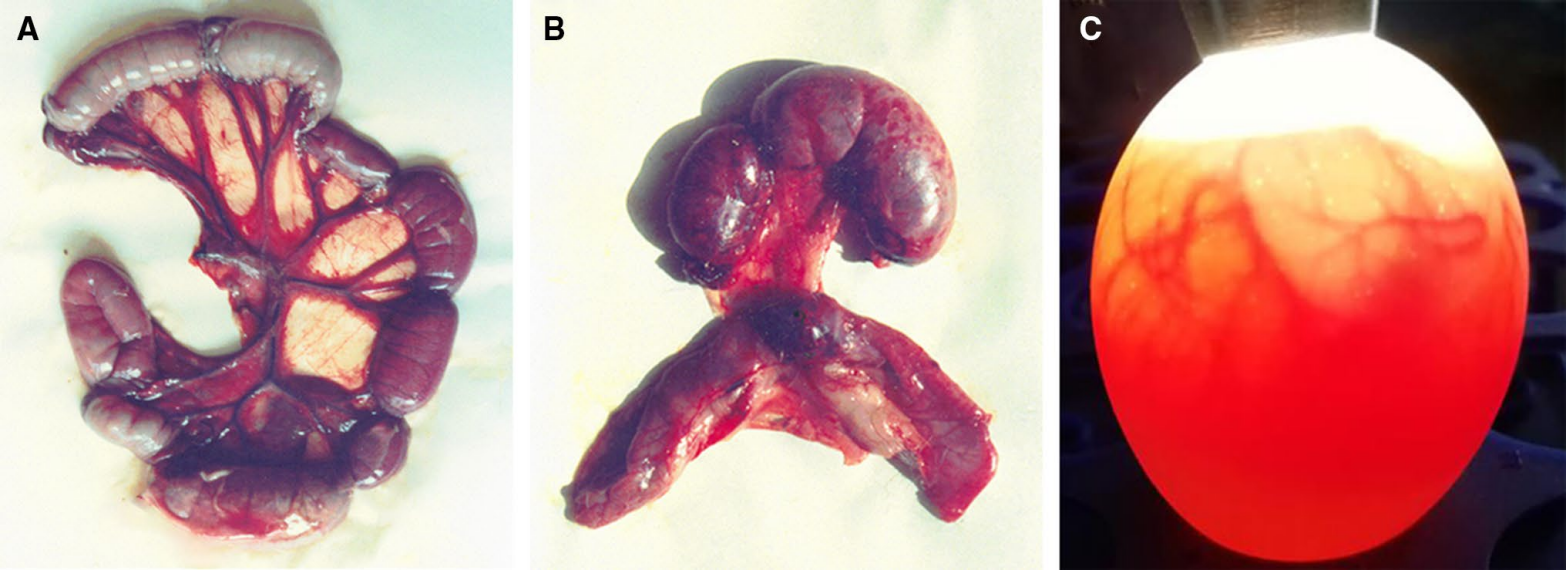
Rabbit ileal loop models and embryonated egg model for resuscitation of the VBNC state. **a** The conventional “four segment ligation of the intestine” rabbit ileal loop model (the rabbit survived for 18–22 h) provided by Huai-Shu Xu (late) and Weishang Ji from Ocean University of China; **b** The improved “double segment ligation of the intestine” rabbit ileal loop model (the rabbit survived for 24–36 h) developed by Huai-Shu Xu and Weishang Ji; **c** Embryonated egg model of seven days old

A variety of animal models (especially the natural host) may be biological mediators for the resuscitation of some bacteria from the VBNC state. For example, the VBNC cells of *Leg. pneumophila* has been reported to be resuscitated in the protozoa *Acanthamoeba polyphaga* (Garcia et al. 2007) and *Acanthamoeba castellanii* (Steinert et al. 1997). VBNC cells of *V. cholerae* were resuscitated to pathogenicity and culturability by their introduction in a rabbit ileal loop model, which was the first animal model to resuscitate VBNC cells (Colwell et al. 1985). The original “four segment ligation of the intestine” rabbit ileal loop model (Fig. 4a) was further modified to “double segment ligation of the intestine” model (Fig. 4b) by Huai-Shu Xu (late; who reported the VBNC state in 1982 for the first time) and Weishang Ji from the Ocean University of China. VBNC cells of *V. cholerae* could also be resuscitated in the intestines of human volunteers (Colwell et al. 1996). In addition, *Cam. jejuni* was resuscitated by the inoculation of VBNC cells into mice (Cappelier et al. 1999). Moreover, the embryonated egg model (Fig. 4c) was successfully used in the resuscitation of bacterial pathogens, *Lis. monocytogenes*, *Edw. tarda* and *Cam. jejuni* from the VBNC state (Cappelier et al. 2007; Du et al. 2007a; Guillou et al. 2008), and this model was more convenient to use than other animal models.

## Implications of VBNC resuscitation stimuli for cultivating uncultured marine microorganisms

The application of molecular biology techniques, especially 16S rRNA gene and metagenomic sequencing, has revolutionized knowledge of bacterial and archaeal diversity in the oceans. This knowledge has helped us to recognize and relate groups of organisms based solely on their genetic sequences. It is well established that less than 1% of the potentially 10^11^–10^12^ microbial species in the natural environment may be grown on laboratory culture media (Hahn et al. 2019; Locey and Lennon 2016); the vast majority of the microorganisms are viable in the environment but they usually do not form visible colonies on agar plates (Kogure et al. 1979; Rappe and Giovannoni 2003). This problem has been described as “The Great Plate Count Anomaly”. Many major divisions of Bacteria and Archaea contain no known cultured species (Castelle and Banfifield 2018). This means that some bacterial and archaeal phyla are known only from sequence data of environmental DNA, and we do not have the cultures of many widely distributed marine phyla.

Laboratory cultures are still immensely beneficial in the era of molecular biology and high-throughput sequencing. Microbial cultures enable detailed studies of cell physiology, genetics and evolutionary relationships, knowing the morphology of the microorganisms, isolation of bacteriophages, and discovery of novel metabolic pathways. It is not always possible to predict the activity of microorganisms and their ecosystem function from genome sequence alone. Having laboratory cultures is essential to understand community-level processes and answering important questions regarding the role of microorganisms in the sea. In addition, metagenomic analysis of microbial communities relies heavily on data obtained from the sequencing of genomes of cultivated species (Carini 2019). Moreover, laboratory cultures are also important for commercially important research, such as screening of natural products. Clearly, cultivation-dependent and cultivation-independent approaches are complementary to the microbial community studies. For the future, it is important to develop new cultivation strategies.

In recent years, some resuscitation stimuli have been used successfully to recover bacteria from natural environments. Rpf protein (Mukamolova et al. 1998, 2002) is one of the most powerful resuscitation stimuli, and a picomole-level concentration could promote the growth of culturable cells by more than 100 times (Su et al. 2013). Ding and Yokota (2010) reported that the addition of a Rpf-containing culture supernatant from *Mic. luteus* could promote the growth of *Curvibacter fontanus* (validation name in Int J Syst Evol Microbiol, 2010, 60:2509–2510); a micro-aerobic organism isolated from well water in Japan. Subsequently, the addition of Rpf-containing culture supernatant from *Mic. luteus* enhanced the isolation of the biphenyl-degrading bacteria from PCB-contaminated soils of e-waste recycling sites in Taizhou, China (Su et al. 2013).

Beside the application of Rpf-containing culture supernatant, the use of recombinant Rpf protein has attracted attention. For example, Ding et al. (2012) demonstrated that the recombinant Rpf protein from *Mic. luteus* had a strong ability to promote the resuscitation of VBNC cells of a high-G + C Gram-positive *Rhodococcus* sp. DS471, which was isolated from soil. Moreover, the introduction of recombinant Rpf protein from *Mic. luteus* enabled the isolation of some unique bacterial species, which belonged to the genera *Arthrobacter*, *Bacillus*, *Bordetella*, *Mycobacterium, Nocardiopsis, Novosphingobium* and *Pandoraea*. Also, Rpf treatment significantly enhanced cellulase activity of the microbial community in mature compost produced from household and agro‐industrial wastes in China (Su et al. 2018). Furthermore, Luo et al. (2019) overexpressed and purified the recombinant Rpf protein from an oil-degrading organism, *Rhodococcus erythropolis*, and showed that Rpf could promote the resuscitation of the VBNC cells of *Rho. erythropolis* as well as efficiently improve the growth of normal *Rho. erythropolis* culture.

Sodium pyruvate was used successfully as a resuscitation stimulus, leading to the recovery of bacteria from natural environments. In this connection, Mu et al. (2018) developed an enrichment culture with a low-nutrient medium containing 10 mmol/L sodium pyruvate for efficiently isolating and culturing previously uncultured bacteria from coastal sediment of China. The work led to the isolation of 97 potentially novel taxa, including one order, one family, 16 genera and 79 species. In addition, it is very common to facilitate the isolation of novel archaeal species by supplementation of sodium pyruvate in the culture medium (Han et al. 2019). Certainly, more chemical stimuli should be applied to recover uncultured bacteria from natural environments in future studies.

## Conclusions

Marine microorganisms exist in an ever-challenging environment, and only a small proportion may be cultivated using currently available techniques. There are a variety of explanations for the inability to culture bacteria in the laboratory, including the use of unsuitable cultivation methods, neglected slow-growing microorganisms, the inability to communicate among microbial cells in pure culture, cell damage induced by oxidative stress of fast-growing bacteria, and the existence of VBNC bacteria. Many Gram-negative bacteria and nonsporulating Gram-positive bacteria can enter the VBNC state, which is a complicated metabolic strategy of bacteria to survive for long-term under adverse conditions. It is assumed that the VBNC response of bacteria is a common process, which may occur in the wider scope of bacterial taxa. In this regard, VBNC cells could constitute a huge reservoir of natural bacteria, which cannot be cultured easily with ordinary cultivation methods. Various resuscitating stimuli, which are able to revert VBNC cells to a metabolically active and culturable state on conventional bacteriological media, have been identified, including physical and chemical stimuli, active proteins, and biological stimuli. However, most studies of VBNC bacteria have focused on pure cultures in the laboratory rather than on environmental bacteria. Stimuli that are currently used on model or indicator strains may well be used to recover cells from the VBNC state in natural environments. Indeed, some resuscitation stimuli have recently been used successfully to recover bacteria from natural environments. Resuscitating indigenous microorganisms from environments may provide a new approach to explore crucial populations, which may play key roles in ecological processes or have great value for industrial applications, and deserve more attention and effort. In addition, different bacterial taxa may adopt different ways to resuscitate, and this point warrants further investigation.
